# DNA-Binding Protein A Is Actively Secreted in a Calcium-and Inflammasome-Dependent Manner and Negatively Influences Tubular Cell Survival

**DOI:** 10.3390/cells13201742

**Published:** 2024-10-21

**Authors:** Gregor Hoppstock, Jonathan A. Lindquist, Antonia Willems, Annika Becker, Charlotte Reichardt, Ronnie Morgenroth, Saskia Stolze, Cheng Zhu, Sabine Brandt, Peter R. Mertens

**Affiliations:** Clinic of Nephrology, Hypertension, Diabetes and Endocrinology, Otto-von-Guericke University Magdeburg, 39120 Magdeburg, Germany; gregor.hoppstock@st.ovgu.de (G.H.); jon.lindquist@med.ovgu.de (J.A.L.); antonia.bock94@t-online.de (A.W.); zhuc111@zju.edu.cn (C.Z.); sabine.brandt@med.ovgu.de (S.B.)

**Keywords:** cold shock domain proteins, danger signal, inflammation, innate immunity, protein secretion

## Abstract

DNA-binding protein A (DbpA) belongs to the Y-box family of cold shock domain (CSD) proteins that bind RNA/DNA and exert intracellular functions in cell stress, proliferation, and differentiation. Given the pattern of DbpA staining in inflammatory glomerular diseases, without adherence to cell boundaries, we hypothesized extracellular protein occurrence and specific functions. Lipopolysaccharide and ionomycin induce DbpA expression and secretion from melanoma and mesangial cells. Unlike its homologue Y-box-binding protein 1 (YB-1), DbpA secretion requires inflammasome activation, as secretion is blocked upon the addition of a NOD-like receptor protein-3 (NLRP3) inhibitor. The addition of recombinant DbpA enhances melanoma cell proliferation, migration, and competes with tumor necrosis factor (TNF) binding to its receptor (TNFR1). In TNF-induced cell death assays, rDbpA initially blocks TNF-induced apoptosis, whereas at later time points (>24 h), cells are more prone to die. Given that CSD proteins YB-1 and DbpA fulfill the criteria of alarmins, we propose that their release signals an inherent danger to the host. Some data hint at an extracellular complex formation at a ratio of 10:1 (DbpA:YB-1) of both proteins.

## 1. Introduction

It has been determined that 20–25% of all genes encode multifunctional proteins [1]. This observation emerged from efforts to identify all gene products, as well as to annotate the molecular function, cellular localization, and participation in biological processes for each protein. The mapping of post-translational modifications, alternative splice products, as well as alternative translational start sites has only added to the complexity of this task. These proteins are often described as pleiotropic, as each possesses a variety of cellular activities that are not necessarily related to one another. Notable among these are transcription factors, such as high mobility group box 1 (HMGB1) and Y-box-binding protein 1 (YB-1), which also occur as secreted proteins [2,3].

Their intracellular activities are clearly distinct from the extracellular. The latter is associated with the induction of inflammatory and immune responses, where they effect cell–cell communication. The secretion of these proteins occurs with tissue damage and in diseases [2,3].

DNA-binding protein A (DbpA) belongs to the Y-box nucleic acid binding proteins. They share a single highly conserved cold shock domain (CSD), which mediates their activities as transcriptional/translational regulators [4]. The Y-box family consists of three members: Y-box-binding protein 1 (YB-1), also known as DNA-binding protein B (DbpB); Y-box-binding protein 2 (DNA-binding protein C (DbpC)/contrin)); and Y box-binding protein 3 (DNA-binding protein A (DbpA), also known as zonula occludens-1 (ZO-1)-associated nucleic acid binding protein (ZONAB) or cold shock domain protein A (CSDA)) [3]. The cold shock domain proteins are found in all organisms from bacteria to humans, with the exception of yeast [5,6,7].

**Figure 1:**
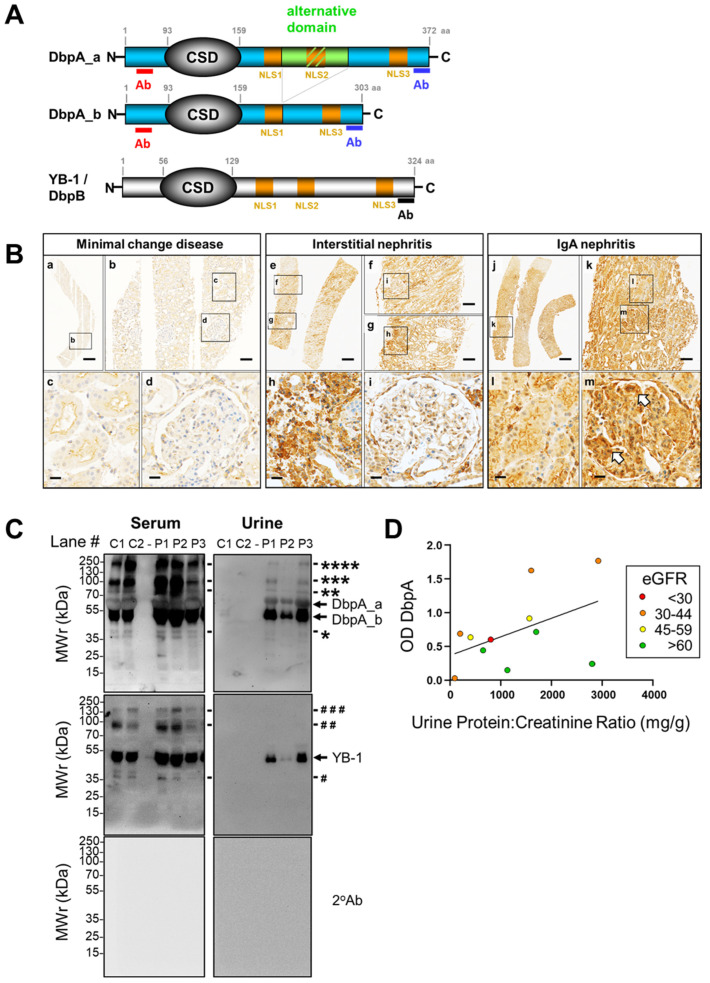
DbpA protein structure and detection of protein in tissue and urine samples. (**A**) Schema depicting the structure of the DbpA isoforms, DbpA_a and DbpA_b, and YB-1/DbpB, respectively. The DbpA isoforms result from alternative splicing of exon 6 and differ by 69 amino acids. The position of the peptides used for polyclonal antibody generation are indicated. Ab, antibody; CSD, cold shock domain; DbpA, DNA-binding protein A; DbpB, DNA-binding protein B; YB-1, Y-box-binding protein 1. (**B**) Immunohistochemistry shows that DbpA expression is barely detectable in the patient with minimal change disease, whereas DbpA is clearly detected within the areas affected by disease, i.e., interstitium in interstitial nephritis biopsies and the mesangial compartment of the glomeruli from IgA nephritis patients. Infiltrating immune cells also appear to show enhanced DbpA expression. Images were made with a Vectra Polaris microscope using a 20× objective. Images were processed using Phenochart software (version 1.0.9). Scale bars: (a) 700 µm, (e, j) 500 µm, (b, f, g, k) 100 µm, (c, d, h, i, l, m) 20 µm. (**C**) DbpA is found in the serum and only in the urine from patients with IgA nephritis, but not from healthy controls. Additional protein bands for DbpA and YB-1 are indicated by the * or # respectively. Ctrl, control; DbpA, DNA-binding protein A; *w*/*o*, without; YB-1, Y-box-binding protein 1. (**D**) Correlation analysis performed by plotting the DbpA content in IgA nephritis patient urine versus the urine protein/creatinine ratio (UPCR). Band intensities determined by Western blotting are presented as optical density (OD). Kidney function (eGFR) is indicated by the color; green dots represent an eGFR > 60 mL/min; yellow, 45–59 mL/min; orange, 30–44 mL/min; and red, <30 mL/min. For most patients, a high DbpA content appears to correlate with reduced kidney function.

The *YBX3* gene containing 10 exons that encode DbpA_a (372 amino acids) is located on chromosome 12 in humans (Figure 1A) [8]. Alternative splicing of exon 6 (Δ69 amino acids, indicated as an alternative domain) results in the formation of a shorter isoform DbpA_b. The protein has a short N-terminal domain rich in proline and alanine residues, an evolutionarily conserved cold shock domain (CSD; residues 93–159), and a long C-terminal domain containing three nuclear localization signals (NLSs), one of which is located within the alternative domain (exon 6). DbpA was first identified via its binding to the epidermal growth factor receptor promoter [9]. It is a component of tight junctions due to its association with ZO-1 [10]. The loss of cell–cell contacts results in the translocation of DbpA from the membrane into the nucleus, where it promotes cell proliferation via the activation of target genes [11].

A characterization of a mouse model of interstitial kidney fibrosis revealed that in *Ybx3* knockout animals, tubular cells exhibit a survival benefit with reduced immune cell infiltration and fibrotic response, suggesting that DbpA fulfills deleterious effects [12]. In line with this is our recent observation that animals lacking DbpA are protected from renal ischemia-reperfusion injury [13].

For YB-1, in addition to localizing in either the cytoplasmic or nuclear compartments, an extracellular occurrence has also been reported [14,15,16]. The secretion of YB-1 requires the acetylation of at least two C-terminal lysine residues by the CBP/p300 acetyl transferase complex and utilizes a secretory pathway involving ABC transporters, as it can be inhibited with glyburide and probenecid [14]. Further, the ubiquitination of YB-1 was reported to be essential for targeting the protein to exosomes, although no ubiquitin was found attached to the extracellular protein [17]. Monomeric YB-1 as well as high molecular weight oligomers and a small fragment designated p18 have all been reported [18,19]. Pro-inflammatory stimuli, such as lipopolysaccharide (LPS), platelet-derived growth factor B (PDGF-B), and transforming growth factor beta (TGF-β), not only induce YB-1 expression, but also its secretion via a non-classical pathway. Extracellularly, YB-1 binds to receptor Notch3 and demonstrates both chemotactic and mitogenic activities [20].

In response to pathogens, infiltrating neutrophils expel their chromatin to form extracellular traps (NETs); this process is known as NETosis [21]. YB-1 was recently shown to be a component of the NETs and its extracellular presence enhanced cytotoxicity [22].

Our initial observation of the non-adherence of DbpA immunostaining towards cell boundaries within tissue samples [23] prompted us to further investigate a pathway for protein secretion.

## 2. Materials and Methods

### 2.1. Human Material

Patients undergoing renal biopsies provided informed written consent in accordance with the principles set out in the Declaration of Helsinki; approval was provided by the Ethics Committee of the Otto-von-Guericke University, Magdeburg, Germany (74/09 and 106/09). Serum and urine samples were collected before biopsy and stored at −80 °C. Ultrasound-guided biopsies were obtained using an 18G needle and fixed in a 4% buffered-formaldehyde solution. For immunohistochemistry, antigen retrieval was performed after rehydration by heating the sample in 10 mM sodium citrate buffer (pH 6.0) in a microwave. Primary antibody staining was performed using affinity-purified rabbit IgG as previously described [23].

### 2.2. Animals

Studies were performed with genetically modified mice on a C57BL/6N or mixed C57BL/6N/J background. Animals were housed according to the FELASA guidelines under specific pathogen-free conditions. All procedures were performed in accordance with the German National Guidelines for the use of experimental animals (AZ UniMD 42502-2-1634 UniMD and KNEP-PME-TWZ-1-23). Bone marrow-dervied macrophages (BMDMs) and primary mouse tubular epithelial cell (PTEC) cultures were prepared as described [13].

### 2.3. Cell Culture

Rat mesangial cells (kindly provided by David H Lovett, San Francisco, CA, USA) and the melanoma A375 cell line (kindly provided by Prof. Birgit Schittek, Tübingen, Germany) were grown in RPMI 1640 medium (Gibco, Paisley, UK) supplemented with 10% FCS (Pan Biotech, Aidenbach, Germany) and 1% Penicillin-Strepomycin (Gibco) at 37 °C and 5% CO_2_ in humidified atmosphere.

### 2.4. DbpA Secretion Assays

Rat mesangial cells were seeded at 2 × 10^5^ cells per well and melanoma A375 cells were seeded at 4 × 10^5^ cells per well in a 6-well tissue culture plate (CellStar, Greiner bio-one, Frickenhausen, Germany) in complete medium. After 48 h, the medium was replaced with 3 mL of fresh starving medium (no FCS) and the cells stimulated with 5 ng/mL LPS (O26:B6 Sigma) or 1 µM ionomycin (Sigma, Schnelldorf, Germany) for the time period indicated. The pathway of protein secretion was determined using specific inhibitors as previously described [14]. Cells were pretreated with the following inhibitors (Calbiochem, Darmstadt, Germany) for 10 min: brefeldin A (10 µg/mL), probenecid (2 µM), glyburide (5 µM), reserpine (20 µg/µL), MCC950 (1 µM), and EGTA (1.5 mM). Following stimulation, the cells and debris were harvested and then pelleted by centrifugation at 400× *g* for 5 min at 4 °C.

### 2.5. Protein Precipitation and Cell Lysis

Following centrifugation, the cell supernatant was collected, and the protein fraction was precipitated by incubation in ice-cold acetone (Carl Roth, Karlsruhe, Germany) (volume 1:2) at −20 °C for >24 h. The precipitated protein was pelleted by centrifugation at 5311× *g* for 30 min at 4 °C. The protein pellet was washed with ice-cold 90% acetone and centrifuged at 17,949× *g* for 30 min at 4 °C. The pellet was air dried and then resuspended in 50 µL of 2x NuPAGE sample buffer (Invitrogen, Dreieich, Germany).

For cell lysates, the mesangial cells were washed with cold PBS and lysed with 500 µL of ice-cold RIPA buffer for 10 min [24]. Cells were then scraped with a rubber policeman, collected in a 1.5 mL tube, and further incubated on ice for 45 min before centrifuging for 15 min at 17,949× *g*, 4 °C. The lysate was collected, and the protein concentration was determined using the Bio-Rad protein assay (Bio-Rad Laboratories, Feldkirchen, Germany). Samples were stored at −80 °C until use.

### 2.6. Immunoblotting

Protein extracts were denatured, separated by NuPAGE, transferred onto PVDF membranes (Carl Roth, Karlsruhe, Germany), blocked with 5% fat-free milk in Tris-buffered saline (1 h, room temperature), and incubated overnight at 4 °C in primary antibodies against YB-1 or DbpA (1:1000) as published [12,14,23]. After washing, the membranes were incubated in secondary antibodies at room temperature for 1 h. The membranes were washed and developed using a chemiluminescent substrate kit Pierce ECL Plus Western blotting substrate (Thermo Fischer, Rockford, IL, USA). Chemiluminescence was detected using the Chemi-Smart system and quantified using LabImage 1D software (version 4.1, INTAS, Göttingen, Germany).

### 2.7. Cell Death Assays

Human kidney proximal tubular epithelial cells (HK-2; ATCC CRL2190) were cultured in Dulbecco’s Modified Eagle Medium (DMEM): Ham’s F-12 medium (1:1) supplemented with 10% heat-inactivated fetal calf serum (Pan Biotech, Aidenbach, Germany) and 1% Penicillin-Strepomycin (Gibco). Cells were treated overnight with 200 µg/mL cycloheximide (CHX) [25]. PTECs were treated overnight with 50 µg/mL CHX. The medium was aspirated and replaced with fresh medium containing CHX and SYTOX Green dye (Invitrogen, Dreieich, Germany) for 30 min according to the manufacturer’s instruction. Prior to stimulation, the cells were imaged using a Keyence BZ-X810 fluorescence microscope to establish the baseline. The number of SYTOX positive cells were then counted over time using the same visual field. *Microvesicle preparation*, *cell migration assays*, and *recombinant protein purification* were performed as previously described [14,26]. Briefly, microvesicles were prepared by sequential centrifugation steps. For cell migration, melanoma A375 cells were grown to confluence before inducing a scratch wound. The cell front was monitored by light microscopy to estimate the rate of wound closure. Mitomycin (10 µg/mL; Sigma) treatment was applied to inhibit cell proliferation. Recombinant Flag-tagged human cold shock proteins were expressed in HEK 293 cells and isolated by immuno-affinity purification.

### 2.8. Statistical Analysis

Statistical analyses using one-way ANOVA were performed using GraphPad Prism 8 software. The data were considered significant when * *p* < 0.05.

## 3. Results

### 3.1. Despite the Absence of a Bona Fide Secretory Motif, Databases on Body Fluid Composition Indicate Extracellular DbpA Occurrence

A phylogenetic analysis of the human cold shock domain protein family shows that amongst the Y-box protein family, YBX1 and YBX3 are more closely related to one another than they are to YBX2 (Uniprot, Supplementary Figure S1). In addition to sharing the evolutionarily conserved cold shock domain (CSD), both proteins also possess three conserved nuclear localization signals (NLSs) (Figure 1A). The subcellular localization prediction system (SherLoc2) suggests that the DbpA protein is primarily cytoplasmic (cytoplasm 0.81, nucleus 0.14, mitochondrial 0.02, peroxisomal 0.02, Golgi apparatus 0.01) [27]. Cross-referencing with the Human Protein Atlas (https://www.proteinatlas.org, accessed on 8 January 2024) confirms a primarily cytosolic localization for the protein. None of the analytic tools tested identified a signal peptide or membrane targeting motif within the N-terminus of the protein [28].

The Human Plasma Proteome database (https://www.hupo.org, accessed on 8 January 2024) reports the concentration of YBX3 from the plasma of healthy individuals to be 230 ng/L. However, as the protein is not predicted to be secreted, it was not analyzed further. Interestingly, the Human Body Fluid Proteome (https://bmbl.bmi.osumc.edu/HBFP, accessed on 8 January 2024) also reports the presence of the YBX3 protein in six body fluids that are milk, plasma/serum, saliva, sweat, tears, and urine (confidence scores of 0.935, 0.81, 0.89, 0.975, 0.94, and 0.89, respectively).

### 3.2. DbpA Expression in Kidney Disease and Secretion by Cells

In tissue from patients with minimal change disease that do not exhibit an inflammatory tissue response, there is a low level of DbpA protein detected in tubular and some glomerular cells. In contrast, immunohistochemistry with kidney tissue from patients with tubulointerstitial nephritis revealed a strong positivity in infiltrating immune cells and kidney resident cells. The most pronounced DbpA staining was detected with biopsies from patients with IgA nephritis showing a strong diffuse staining throughout the tubular and glomerular compartments. For the glomerular cells, positivity was pronounced within the mesangial compartment (Figure 1B, indicated by arrowheads). Again, protein expression patterns are not restricted to cell boundaries.

To test for DbpA secretion, serum and urine samples were collected from healthy controls and IgA nephritis patients, separated by electrophoresis, and immunoblotted (Figure 1C). Probing of the membranes with antibodies specific for YB-1 or DbpA confirmed the presence of both proteins in all serum samples tested. On the other hand, the proteins were only detected in the urine samples from IgA nephritis patients. This may indicate a spill-over effect with proteinuria. However, a simple correlation between DbpA content and the degree of proteinuria was not found when analyzing more samples (Supplemental Figure S2), suggesting a more complex relationship between DbpA secretion and disease. A linear relationship was observed between the content of DbpA and YB-1 in serum by ELISA and urine by immunoblotting, suggesting that their secretion is somehow related. Plotting the urinary DbpA content versus the urine protein/creatinine ratio (UPCR), an indicator of kidney disease severity (Figure 1D), was performed. Patients with ‘normal’ kidney function are shown with green dots (eGFR > 60 mL/min), whereas yellow, orange, and red dots indicate decreasing kidney function (45–59, 30–44, <30 mL/min, respectively). Thus, the highest amount of DbpA was detected in those samples with progressive IgA nephritis and decreased kidney function.

**Figure 2:**
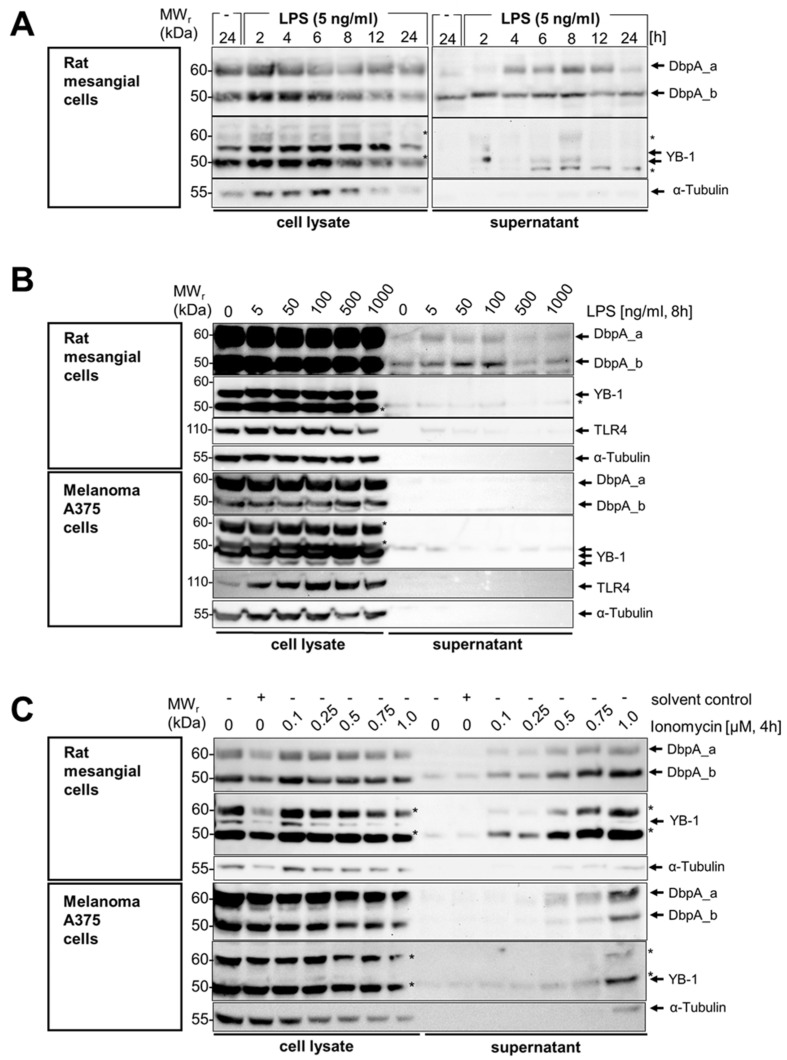

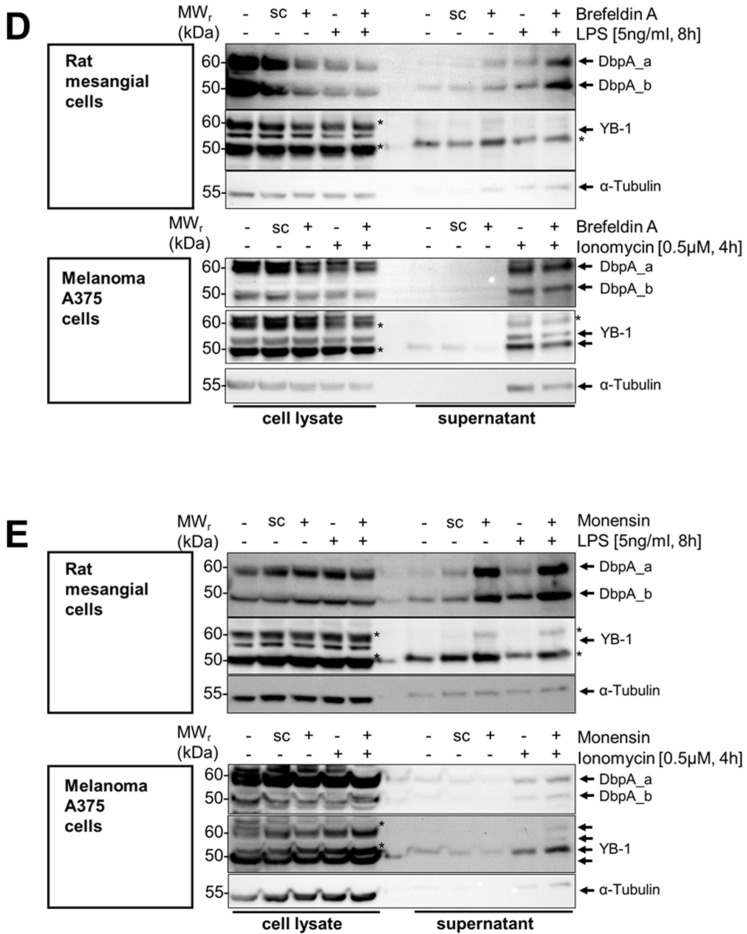
Time- and concentration-dependent DbpA secretion following LPS and ionomycin stimulation of rat mesangial and A375 melanoma cells. (**A**) An LPS concentration-dependent DbpA secretion takes place in rMCs. While DbpA_b is constantly secreted, DbpA_a appears around 4 h and then disappears after 12 h. (**B**) DbpA is secreted in a concentration-dependent manner by rMCs with a maximum at 100 ng/mL LPS for 8 h. Melanoma A375 cells do not secrete DbpA in response to LPS stimulation. (**C**) Both rMCs and A375 show a concentration-dependent secretion of DbpA_a/b following ionomycin stimulation for 4 h. (**D**) DbpA secretion in rat mesangial cells (rMCs) following LPS incubation is enhanced after preincubation with Brefeldin A, an inhibitor of the classical secretory pathway. In melanoma A375 cells, brefeldin A preincubation before ionomycin stimulation has no effect on DbpA secretion. (**E**) Monensin, another inhibitor of the classical secretory pathway, induces DbpA secretion in rMCs and enhances the amount of secreted DbpA when combined with LPS. DbpA secretion in response to ionomycin stimulation is not reduced in melanoma A375 cells preincubated with monensin. Each experiment was performed at least 2 times. The asterisks indicate bands from the previous antibody. DbpA, DNA-binding protein A; LPS, lipopolysaccharide; rMCs, rat mesangial cells; sc, solvent control; YB-1, Y-box-binding protein 1.

Given these initial observations, we set up an in vitro system with rat mesangial cells that were challenged with LPS (5 ng/mL) in a serum-free medium from 2 to 24 h (Figure 2A). Cell lysates were prepared, and the corresponding supernatants were collected and precipitated by the addition of ice-cold acetone. Following immunoblotting, the cell lysates show a stimulus-induced increase in cold shock protein expression. An analysis of the supernatants shows a time-dependent secretion of the DbpA_a protein that appeared between 4 and 12 h of LPS incubation, whereas the DbpA_b protein shows a steady state release. YB-1 protein secretion, on the other hand, shows an initial peak at 2 h and a second peak coinciding with that of DbpA_a. Tubulin, a cytosolic protein, was not released during this incubation and served as a control for cell lysis and death. For DbpA_b, there appears to be a constitutive protein release into the supernatant even in the absence of LPS. The apparent molecular weight of the secreted DbpA_b isoform in stimulated cells appears slightly higher (~2 kDa) than that of the cytosolic protein, suggesting a post-translational modification.

Next, we wished to narrow down the concentration-dependency of the LPS effect on DbpA secretion after 8 h of stimulation. We chose to repeat the experiments with mesangial cells as well as melanoma A375 cells, for which YB-1 secretion has been reported (Figure 2B) [15]. There was a concentration-dependency of DbpA_a and DbpA_b secretion with the most pronounced secretion in rat mesangial cells at 5 to 100 ng/mL, whereas no secretion of either DbpA isoform was observed in the melanoma A375 cell line (compare Figure 2B upper and lower panels). For YB-1, little or no secretion was observed in response to LPS. An upregulation of TLR4 expression detected in the melanoma A375 cell lysate served as a control for effective LPS stimulation and cellular responsiveness. Similarly, we chose to incubate both cell lines with the calcium ionophore ionomycin, a known secretagogue. Here, a clear dose-dependent secretion of DbpA_a and DbpA_b was detected in both cell lines (Figure 2C, upper and lower panels). Notably, rat mesangial cells secrete more DbpA_b than DbpA_a, whereas the opposite was detected with melanoma A375 cells. In both cases, a subtle shift in the relative molecular weight was seen in the secreted proteins (~2 kDa), compare the 24 h unstimulated sample with 2 h LPS.

### 3.3. DbpA Is Secreted by a Non-Classical Secretion Pathway

To characterize the mechanism(s) underlying DbpA secretion, we first tested the classical protein secretion pathway. Here, we inhibited the endoplasmic reticulum-Golgi apparatus using brefeldin A and monensin. For rat mesangial cells, the addition of brefeldin A alone induced a weak secretion of DbpA, which was markedly enhanced in combination with LPS (Figure 2D top panel). The DbpA content of the cell lysate is markedly reduced in response to the incubation, suggesting that either DbpA secretion or degradation is enhanced. For the melanoma A375 cell line, brefeldin A has no effect upon ionomycin-induced DbpA secretion. The slight increase in the presence of cytosolic protein α-tubulin in the brefeldin A-challenged melanoma A375 cells may indicate a toxic effect of this compound.

Similar to brefeldin A, monensin incubation markedly enhanced DbpA secretion in rat mesangial cells even without LPS stimulation (Figure 2E). Monensin showed little or no effect on DbpA secretion in ionomycin-challenged melanoma A375 cells. Taken together, the data suggest that the pathway(s) of DbpA secretion are cell-specific.

**Figure 3:**
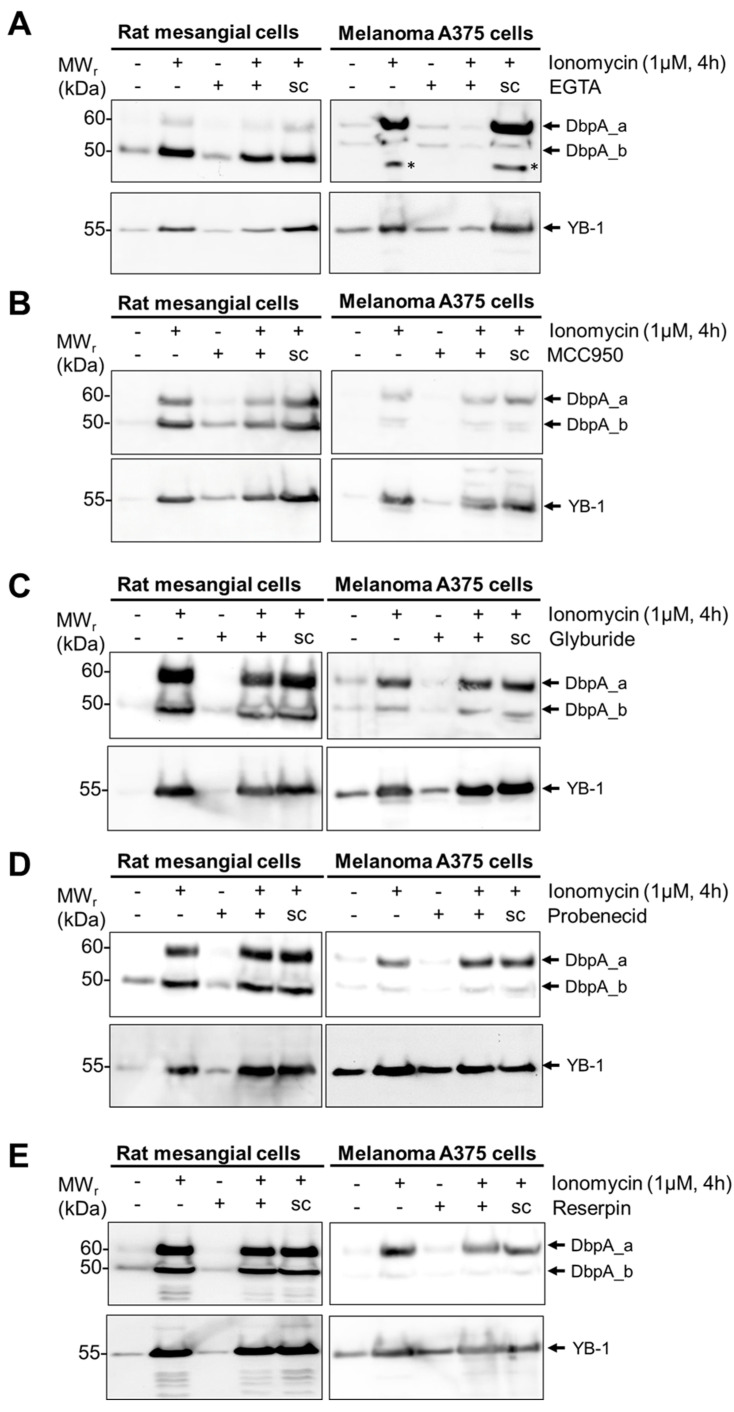
DbpA secretion requires inflammasome activation. (**A**) rMCs and melanoma A375 cells were either left untreated or were pretreated with inhibitor EGTA (1.5 mM) or solvent (sc), as indicated, before stimulating the cells with ionomycin for 4 h. Cell supernatants were collected, acetone precipitated, and then separated by gel electrophoresis. The presence of extracellular DbpA or YB-1 is determined by immunoblotting using the antibodies indicated. Recombinant proteins were included to determine the position of the proteins (see Supplementary Figures S3 and S4). Each experiment was performed at least 2 times. *, Nonspecific band. (**B**) MCC950 (1 µM). (**C**) Glyburide (5 µM). (**D**) Probenecid (2 µM). (**E**) Reserpine (20 µg/µL). DbpA, DNA-binding protein A; LPS, lipopolysaccharide; rMCs, rat mesangial cells; sc, solvent control; YB-1, Y-box-binding protein 1.

Since numerous alternative protein secretion pathways exist [29], we tested a panel of inhibitors targeting specific mechanisms. As both cell lines secrete DbpA in response to ionomycin stimulation, we focused on this stimulus. As expected, the incubation of cells with EGTA, which chelates extracellular calcium, completely abolished the stimulus-dependent secretion of DbpA_a in both cell lines (Figure 3A). However, in rMCs, the stimulus-dependent secretion of DbpA_b appears unaffected by EGTA suggesting that this may involve calcium-independent mechanisms [30]. The incubation of cells with MCC950, an NLRP3-inflammasome inhibitor, resulted in a 50–70% reduction in the stimulus-dependent secretion of DbpA_a and a 30–50% reduction in DbpA_b secretion for both cell lines (Figure 3B and Supplementary Figure S3). Since calcium influx activates the inflammasome [31], these results agree, suggesting that DbpA is secreted via gasdermin D pores. The application of glyburide, probenecid, or reserpine showed no effect on the stimulus-induced secretion of DbpA (Figure 3C–E). The ability of brefeldin A and monensin to enhance DbpA secretion fits the gasdermin pathway, as both compounds are known to enhance IL-1β secretion [32,33]. The results of the inhibitor experiments are summarized in Table 1.

**Figure 4:**
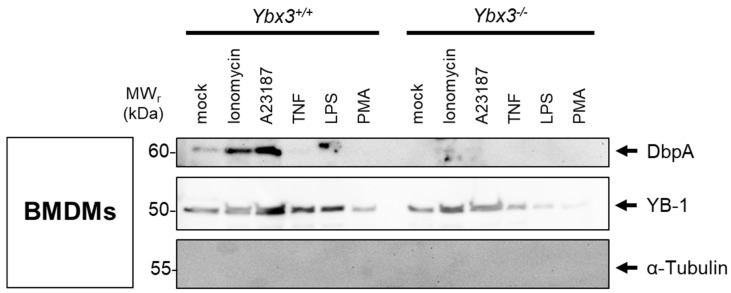
Cold shock proteins are secreted independently of one another. Murine bone-marrow-derived macrophages (BMDMs) from wild-type (*Ybx3*^+/+^) and *Ybx3*-deficient (*Ybx3*^−/−^) mice were kept in culture or challenged with the indicated stimuli. Cell supernatants were precipitated with acetone, separated on polyacrylamide gels, and transferred onto membranes. Cold shock protein content was determined by probing with the indicated antibodies. The cytoskeletal protein α-tubulin was included to control for cell lysis.

To determine whether the cold shock proteins YB-1 and DbpA are secreted independently or as a complex, primary bone marrow-derived macrophages (BMDMs) were generated from wild-type and *Ybx3*-deficient mice. Following challenge with pro-inflammatory stimuli (Figure 4), the cold shock protein content in the medium was determined by immunoblotting. A basal level of cold shock protein is observed in the supernatant of cultured cells (mock). Challenge of wild-type cells with the calcium ionophores ionomycin or A23187, as well as LPS, induce a clear secretion of DbpA, whereas TNF and PMA do not. As expected, no DbpA protein is detected in the medium of *Ybx3*-deficient cells. Similar to Figure 2A, the DbpA protein secreted in response to LPS shows a slight increase in relative molecular weight (~2 kDa). Most pro-inflammatory stimuli tested also induce the secretion of YB-1, which also takes place in *Ybx3*-deficient cells. These data support our observation from the inhibitor studies that cold shock protein secretion occurs independent from each other.

**Figure 5:**
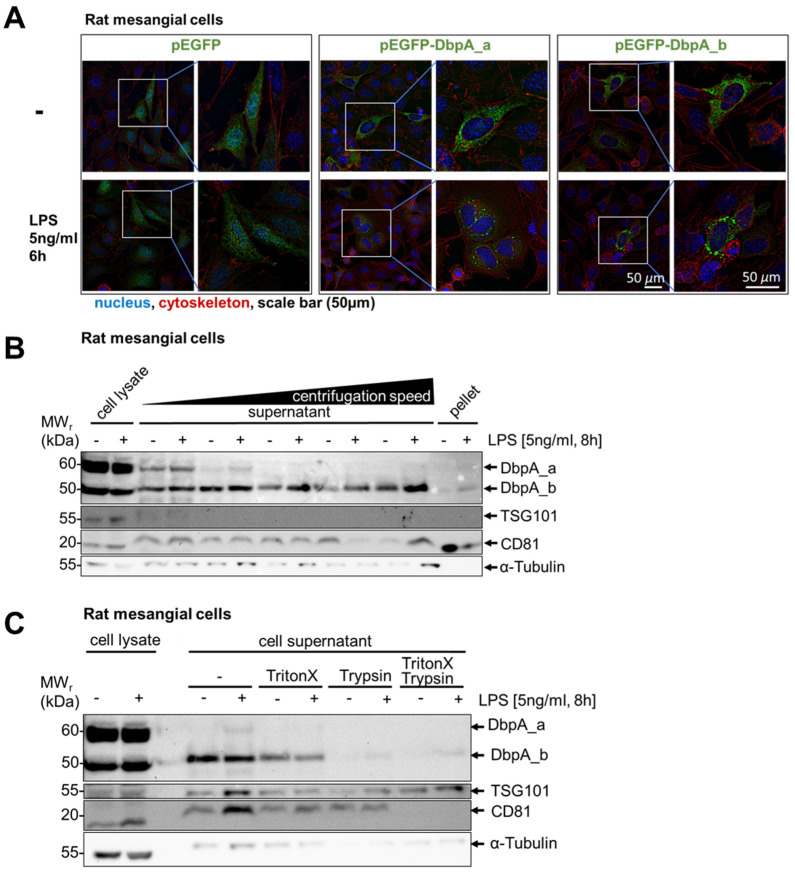
DbpA is released as a non-vesicular protein. (**A**) Fluorescence microscopy revealed GFP-DbpA_a and DbpA_b enriched vesicles in rMCs, transfected with both GFP-DbpA_a and DbpA_b, followed by LPS stimulation for 6 h. No vesicles were detected in GFP-transfected control cells. Scale bar is 50 µm. (**B**) Isolation of the exosomal fraction from LPS-treated rMC supernatants by differential ultracentrifugation showed that no DbpA_a and only a tiny amount of DbpA_b was found in the exosomes, suggesting that DbpA secretion does not involve membrane vesicles. (**C**) Supernatants of LPS-treated rMCs were incubated with the detergent Triton X100, which may disrupt lipid vesicles, and/or the protease trypsin. Trypsin alone digested DbpA, showing that DbpA is not protected by vesicular structures and is released as a non-vesicular protein. Thus, we conclude that DbpA is not present in exosomes. Each experiment was performed at least 2 times. DbpA, DNA-binding protein A; GFP, green fluorescent protein; LPS, lipopolysaccharide; rMCs, rat mesangial cells; TSG101, tumor susceptibility gene 101.

Next, we visualized DbpA to determine whether changes in its subcellular localization occur upon stimulation (i.e., in vesicular structures). To this end, rat mesangial cells were transfected with either GFP alone or a GFP-tagged DbpA_a or DbpA_b constructs. Transfected cells were left untreated or stimulated with LPS for 6 h, as secretion was maximal at 8 h. In unstimulated cells, GFP protein alone showed a diffuse pattern, whereas the GFP-tagged DbpA proteins were primarily cytosolic (Figure 5A). Following LPS stimulation, no change in subcellular localization was observed for the GFP protein, whereas cells with GFP-tagged DbpA_a or DbpA_b formed large vesicular bodies (Figure 5A).

To determine the nature of these vesicles, the supernatants from both untreated and LPS-stimulated rat mesangial cells were subjected to differential centrifugation steps (Figure 5B). Staining of the tetraspan molecule CD81 and multivesicular body protein tumor susceptibility gene 101 (TSG101) revealed a successful enrichment of exosomes during the fractionation procedure. However, no DbpA_a and only minute amounts of DbpA_b protein were visible in the CD81-enriched exosomal pellets, suggesting that the DbpA isoforms are present in larger structures that are not exosomes. To investigate the nature of these structures, cell supernatants were subjected to treatment with detergent and/or protease (Figure 5C). While Triton X100 treatment had little effect on the secreted DbpA protein, trypsin completely ablates its detection, suggesting that DbpA is present in a membrane-free protein complex that is degraded by trypsin. Given that the N- and C-terminal domains of the cold shock proteins exist as intrinsically disordered regions, this may allow these proteins to dynamically form self-assembled membrane-less organelles, such as stress granules and P-bodies [3,34,35,36]. This agrees with a previous report showing YB-1 secretion via stress granules [3,16].

### 3.4. Extracellular DbpA Stimulates Migration and Proliferation

**Figure 6:**
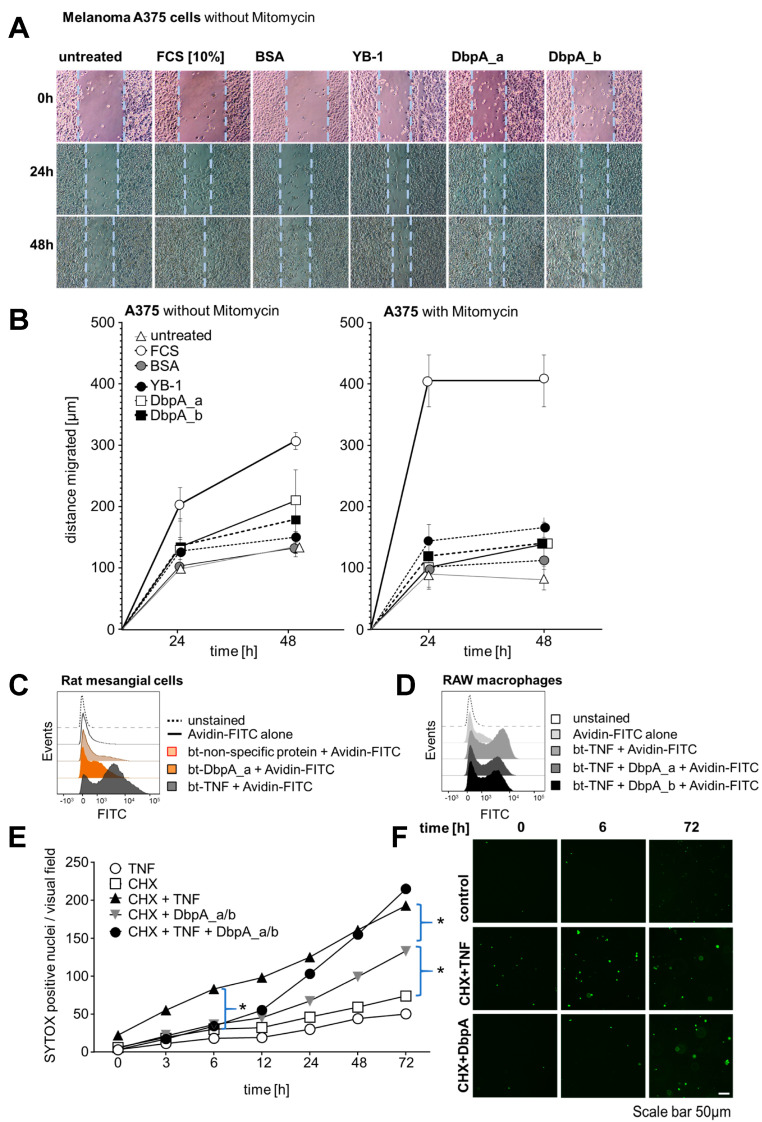

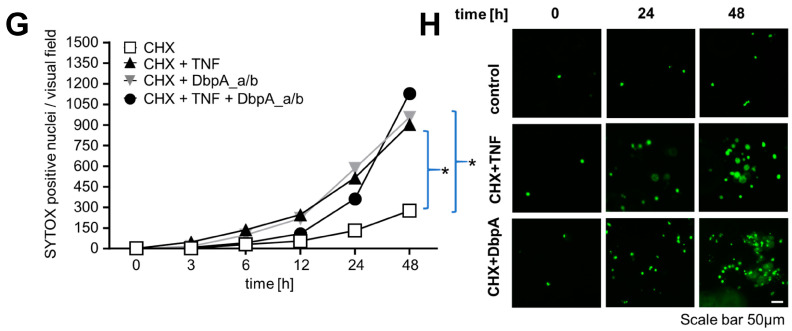
Extracellular DbpA promotes cell migration, binds to cell membranes, and delays cytotoxic TNF effects. (**A**) The wound closing capacity of melanoma A375 cells, incubated with and without mitomycin, an inhibitor of cell proliferation, shows enhanced wound closure following application of recombinant cold shock proteins (YB-1, DbpA_a, and DbpA_b (each 1 µg/mL)). BSA (1 µg/mL, negative control), FCS (10%, positive control). (**B**) Quantification of cell migration in response to the stimuli shown in (**A**) is presented. (**C**) Biotinylated recombinant human DbpA demonstrates cell surface binding on rMCs. An unspecific biotinylated protein was included as the negative control and biotinylated TNF as the positive control. (**D**) Recombinant human DbpA interferes with the binding of biotinylated TNF to RAW macrophages. Avidin–FITC alone was included as the negative control and biotinylated TNF as the positive control. (**E**,**G**) DbpA modifies the death-inducing activity of TNF. HK-2 cells (**E**) or mouse PTECs (**G**) were either left untreated or incubated overnight with cycloheximide (CHX). Cells were then washed, SYTOX green was applied, and the cells were treated as indicated. Cell death was determined by the number of SYTOX positive nuclei appearing over time. (**F**,**H**) Data were analyzed using one-way ANOVA. * *p* < 0.05. Scale bar is 50 µm. Each experiment was performed at least 3 times. BSA, bovine serum albumin; CHX, cycloheximide; DbpA, DNA-binding protein A; HK-2, human kidney 2; TNF, tumor necrosis factor; PTEC, primary tubular epithelial cell; YB-1, Y-box-binding protein 1.

The occurrence of extracellular DbpA raises the question of its functional relevance. Intracellular DbpA promotes proliferation by regulating the expression of cyclin D1 and proliferating cell nuclear antigen (PCNA) [37,38]. Therefore, we studied the proliferative response of cell lines upon stimulation with extracellular DbpA. Changes in proliferation as well as motility were assessed in scratch-wound assays, in which the cell monolayer is ‘injured’. Melanoma A375 cells were grown to confluency and a scratch was introduced using a rubber policeman. Recombinant human DbpA_a or DbpA_b proteins (1 μg/mL) had a stimulatory effect comparable to that observed with recombinant YB-1 (Figure 6A). The addition of bovine serum albumin (BSA) showed no benefit as the rate of wound closure was similar to that of untreated cells. The addition of recombinant DbpA_a or DbpA_b proteins for 24 h increased the proliferation rate. The addition of fetal calf serum (FCS) served as the positive control. Quantification is provided in Figure 6B (left panel).

In a similar approach, melanoma A375 cell migration was monitored by light microscopy for 24 and 48 h under non-stimulated conditions and after the addition of rDbpA. Here, cell proliferation was blocked by the addition of mitomycin C before cell stimulation to monitor cell migration exclusively (Figure 6B, right panel). rDbpA marginally increased cell migration when compared with the basal migration rate under unstimulated conditions. FCS served as the positive control.

In summary, extracellular DbpA exerts a potent proliferative response on melanoma A375 cells and promotes cell migration. Since the observed effects of both DbpA_a and DbpA_b are similar to that of YB-1, we hypothesized that one or more conserved domains probably mediate this activity. Given the striking similarities between DbpA secretion and other non-classically secreted nuclear proteins (HMGB1, S100A, and YB-1), one might speculate that the active secretion of highly conserved nuclear proteins reflects an evolutionarily conserved mechanism to induce inflammation and cell activation [39].

To further investigate the functional relevance of extracellular DbpA, we performed cell surface binding assays using biotinylated recombinant DbpA_a protein. We reckoned that if this protein is secreted, then it might also have a receptor. When we utilized rMCs for the binding assay, the addition of FITC-labelled avidin alone showed no binding to the cells (Figure 6C). The addition of a biotinylated control protein resulted in minimal non-specific binding, whereas the addition of biotinylated DbpA_a yielded a shift in the binding profile similar to that observed for biotinylated tumor necrosis factor (TNF; serving as the positive control).

As we have recently shown that extracellular YB-1 competes with TNF for binding to TNF receptor 1 (TNFR1) [26], we performed analogous TNF receptor binding assays using murine RAW macrophages (Figure 6D). The addition of FITC-labelled avidin alone resulted in minimal binding to the cells when compared to the addition of biotinylated TNF (btTNF; positive control). The addition of recombinant DbpA_a or DbpA_b reduced btTNF cell binding by 20–30%, suggesting that both isoforms are able to compete with TNF for receptor binding.

Given that proximal tubular cells are TNF-responsive [40], we next explored cell death assays using renal tubular epithelial cells in a classical ‘two-hit’ model (Figure 6E,F) [41]. The incubation of an immortalized human proximal tubule epithelial cell line (HK-2) with TNF or cycloheximide (CHX) alone showed no effect, whereas the addition of TNF together with CHX clearly promoted cell death, beginning already at 3 h and steadily increasing over time. The addition of DbpA_a/b (1:1, similar to stimulated rMCs) initially had no effect; however, beginning at 12 h, a noticeable increase in cell death occurred. Similarly, the combination of DbpA_a/b and TNF initially showed no difference compared to CHX challenged cells, suggesting that DbpA may prevent TNF signaling. However, similar to DbpA_a/b alone, an increase in cell death was noticeable after 12 h. This response appeared to overtake TNF by 48 h. We observed a similar response in primary mouse tubular epithelial cells (PTECs) (Figure 6G,H).

Thus, short-term DbpA secretion is beneficial to tubular cells by inducing cell migration and delaying TNF-induced cell death. However, a prolonged exposure of cells to DbpA and TNF is detrimental. TNF receptors signal as trimers or higher order oligomers on the cell surface [42]; it is possible that DbpA influences either the stoichiometry of the receptor or its rate of internalization.

## 4. Discussion

**Figure 7:**
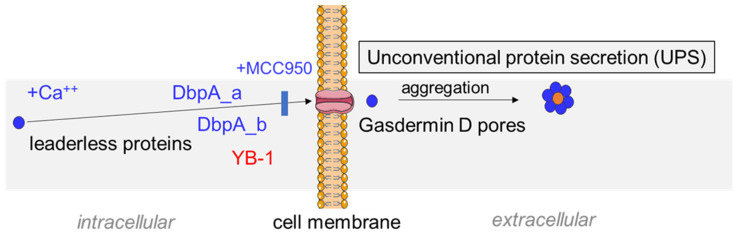
Scheme depicting the mechanism of ionomycin-induced cold shock protein (CSP) secretion. Ionomycin triggers intracellular calcium release that activates the inflammasome, which in turn, facilitates the secretion of intracellular cold shock proteins. The inhibition of either calcium influx or inflammasome activation blocks stimulus-induced protein secretion. The analysis of *Ybx3*-deficient cells shows that cold shock protein secretion occurs independent of one another. Taken together, our data suggest that both proteins aggregate in the serum to form a complex, which is also found in the urine following kidney injury. Since cold shock proteins bind RNA and DNA, we hypothesize that extracellular RNA/DNA may act as a stabilizing factor that explains the observed stoichiometry of 10 DbpA per 1 YB-1 molecule.

This is the first report defining cold shock protein DbpA as an actively secreted protein. Our detailed analysis of different cell lines as well as primary cell cultures provides a complex picture on the regulation of cell type-specific protein secretion. Notably, the active secretion mode is distinct from those previously reported for YB-1 [14,15,16,20]. Our data with inhibitors of unconventional protein secretion pathways, as well as the analysis of cells for vesicular structures that may serve as secretory vehicles, indicate a mechanism linked to inflammasome activation and gasdermin D pores (Figure 7).

We hypothesize that DbpA fulfills a prominent extracellular role in inducing immune cell recruitment and resolving inflammation at later time points by enhancing cell death through cytotoxic effects. One of the most important findings relates to the formation of extracellular DbpA:YB-1 protein complexes. Results from the analysis of serum and urine samples, by ELISA and Western blotting, respectively, identified a linear relationship with a stoichiometry of approximately 10:1. Both proteins possess intrinsically disordered regions that may contribute to their aggregatory potential [43]; however, the role of cofactors, such as extracellular RNA/DNA cannot be excluded. Future studies will be needed to address the obvious questions of when and how this complex formation is orchestrated, what determines its degradation and half-life, as well as whether cofactors are required for the multimerization of both proteins. The latter question also relates to the observed formation of high molecular weight aggregates. One may speculate that extracellular cold shock protein interactions with cell surface receptors are fine-tuned through pro- and anti-aggregatory scenarios.

Alarmins are endogenous danger signals, exemplified by HMGB1 [39,44], which are released from cells undergoing non-apoptotic cell death but retained within cells during apoptosis. Immune cells can also be induced to secrete these proteins using specialized secretion pathways. Once released, alarmins recruit and activate the receptor-expressing cells of the innate immune system. They also induce the proliferation and migration of tissue cells in order to facilitate repair, thus restoring homeostasis.

Our recent study, characterizing a murine model of interstitial fibrosis using *Ybx3* knockout mice, showed enhanced tubular cell survival, a reduced infiltration of immune cells into the damaged kidney, as well as a reduced fibrotic response in the knockout animals [12]. The phenotype observed in these mice agrees with the role of an alarmin.

One limitation is that we study isolated stimuli, whereas in the context of disease, injured cells may be releasing a multitude of DAMPs. The ability of these stimuli to cross-talk or prime other signaling pathways is just only the beginning to be investigated.

Further work is required to determine how the presence of extracellular DbpA correlates with disease activity. The presence of DbpA in the serum of both healthy and diseased individuals questions the cellular source of this protein. DbpA’s homolog, YB-1, was recently demonstrated to be released as a component of the neutrophil extracellular traps [22]. This raises the question as to whether DbpA similarly promotes NETosis.

Here, we have shown that DbpA is released from living cells in response to specific stimuli using specialized secretions systems (Figure 7). The extracellular protein appears to activate receptor-expressing cells and promote tissue repair in order to restore homeostasis. We hypothesize that their release signals an inherent danger to the host and may play a central role in the induction of inflammation.

## 5. Conclusions

Here we report a novel aspect of DbpA activity as a secreted protein. A linear relationship between extracellular DbpA and YB-1 content in the serum and urine of patients was identified, suggesting that these proteins form aggregates with a stoichiometry of 10:1 (DbpA:YB-1). Having recently shown that *Ybx3*-deficient mice are protected in animal models of kidney disease [12,13], it now remains to be determined to what extent extracellular DbpA contributes to disease activity and whether targeting extracellular DbpA may be a means to slow the development of fibrosis and thereby prolong kidney function.

## Figures and Tables

**Table 1:** Summary of the inhibitor experiments. Black arrow indicates enhanced protein secretion, horizontal bar indicates no change, and red arrow indicates a reduced secretion. As melanoma A375 cells did not secrete cold shock protein in response to LPS stimulation, no inhibitor experiments were performed. BFA, brefeldin A; DbpA, DNA-binding protein A; EGTA, ethylene glycol-bis(β-aminoethyl ether)-N,N,N′,N′-tetraacetic acid; LPS, lipopolysaccharide; rMCs, rat mesangial cells; YB-1, Y-box-binding protein 1.

				Inhibitors						
Cell Line	Stimulus	Protein Secretion		BFA	Monensin	EGTA	MCC950	Glyburide	Probenecid	Reserpin
rMC	LPS	DbpA_a	Yes	⬆	⬆	Not tested				
		DbpA_b	Yes	⬆	⬆					
		YB-1	No	⚊	⚊					
	Ionomycin	DbpA_a	Yes	⚊	Not tested	⬇	⬇	⚊	⚊	⚊
		DbpA_b	Yes	⚊		⚊	⬇	⚊	⚊	⚊
		YB-1	Yes	⚊		⬇	⬇	⚊	⚊	⚊
A375	LPS	DbpA_a	No	No secretion, therefore not tested						
		DbpA_b	No							
		YB-1	No							
	Ionomycin	DbpA_a	Yes	⚊	⚊	⬇	⬇	⚊	⚊	⚊
		DbpA_b	Yes	⚊	⚊	⬇	⬇	⚊	⚊	⚊
		YB-1	Yes	⚊	⬆	⬇	⬇	⚊	⚊	⚊

## Data Availability

Inquiries should be directed to the corresponding author.

## Supplementary Materials

The following supporting information can be downloaded at: https://www.mdpi.com/article/10.3390/cells13201742/s1, Figure S1: Phylogenic analysis of the cold shock domain protein family; Figure S2: Correlation analyses; Figure S3: Titration of the inflammasome inhibitor MCC950; Figure S4: Purification of recombinant human DbpA_a and DbpA_b proteins.
