# Supplementary material for: DNA-Binding Protein A Is Actively Secreted in a Calcium-and Inflammasome-Dependent Manner and Negatively Influences Tubular Cell Survival
Source: Cells. 2024 Oct 21;13(20):1742. doi: 10.3390/cells13201742 (PMC11506473; doi:10.3390/cells13201742)
Supplement: Supplementary file 1 [file cells-13-01742-s001.zip › cells-3221631-supplementary.pdf]

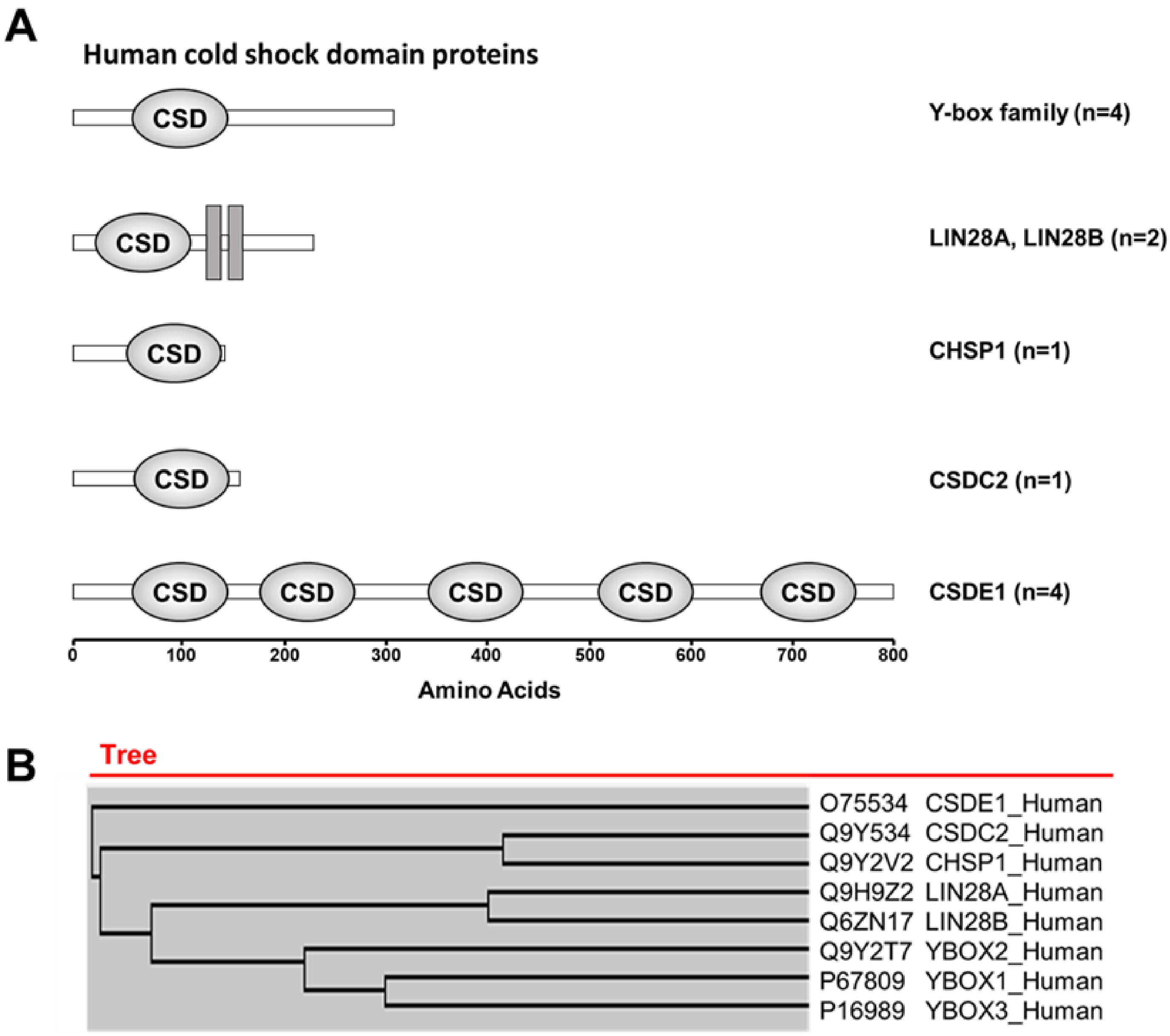

Figure S1. Phylogenic analysis of the cold shock domain protein family. (A) Schema depicting the structure of the cold shock domain (CSD) protein family. The grey boxes indicate zinc-finger domains unique to LIN28. (B) Results of the phylogenic analysis performed using the Uniprot software on the indicated sequences.

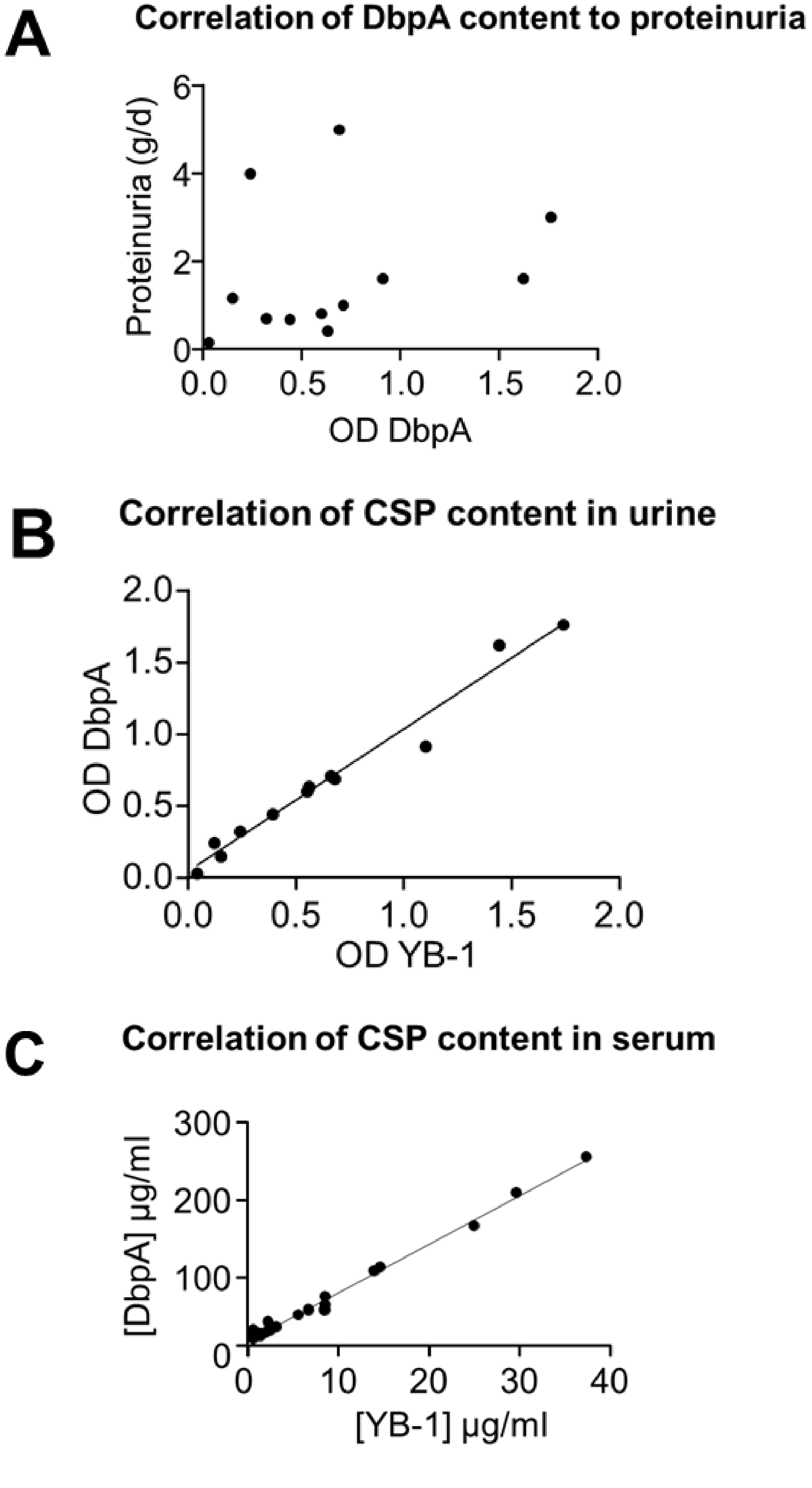

Figure S2. Correlation analyses. (A) Plotting the degree of proteinuria in IgA nephritis patients *versus* the amount of DbpA in the urine shows no correlation. (B) Plotting the amount of DbpA *versus* the amount of YB-1 in patient urine by western blot shows a striking linear correlation. (C) Plotting the amount of DbpA *versus* the amount of YB-1 in serum by ELISA also shows a linear correlation with a stoichiometry of ~10:1.

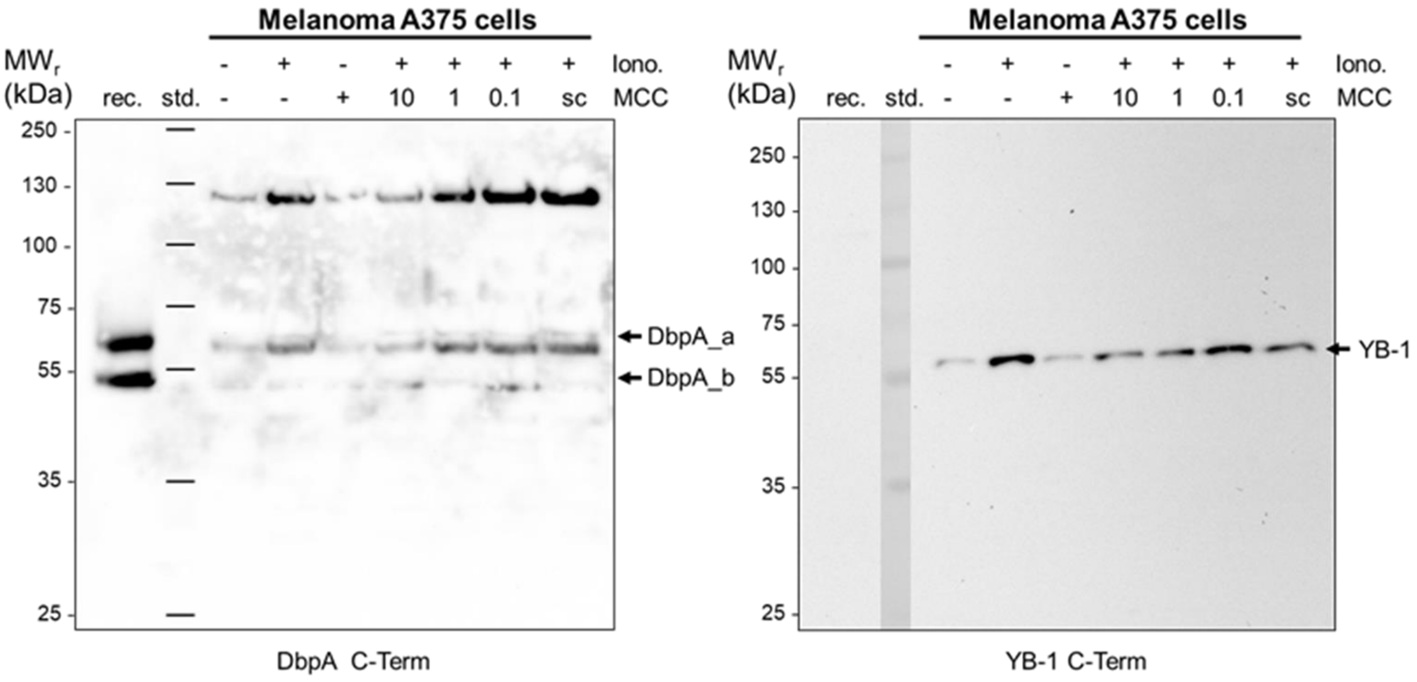

Figure S3. Titration of the inflammasome inhibitor MCC950. Melanoma A375 cells were either left untreated (-) or stimulated with Ionomycin 1μM (+) with or without the application of the inflammasome inhibitor MCC 950 at the concentration indicated (in μM). A band appears at 130 kDa, as well as the expected DbpA_a and DbpA_b bands at 65 and 50 kDa respectively. A375 cells show a dose-dependent inhibition of protein secretion in response to MCC application. Membranes were probed with the antibodies indicated. DbpA, DNA-binding protein A; rec, recombinant protein; sc, solvent control; std, standard; YB-1, Y-box-binding protein 1.

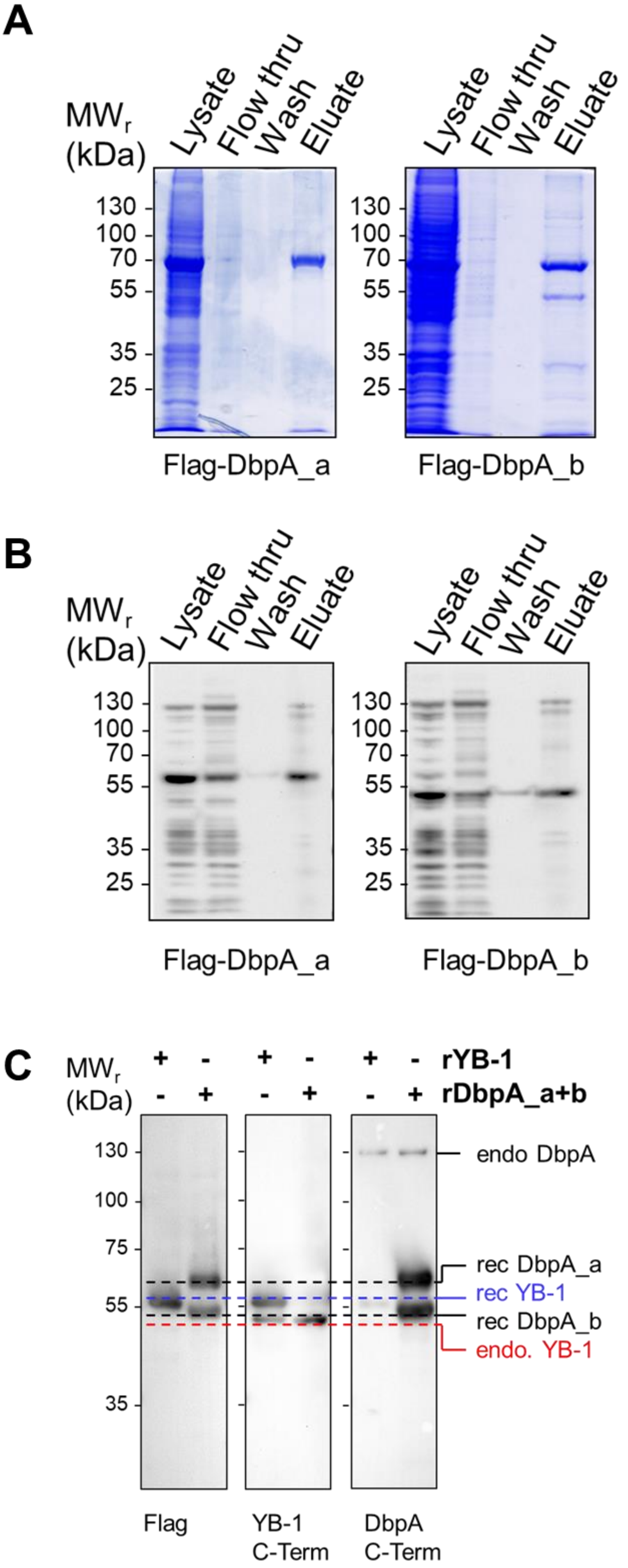
A
MWr (kDa)
Lysate
Flow thru
Wash
Eluate
130
100
70
55
35
25
Flag-DbpA_a
Flag-DbpA_b
B
MWr (kDa)
Lysate
Flow thru
Wash
Eluate
130
100
70
55
35
25
Flag-DbpA_a
Flag-DbpA_b
C
MWr (kDa)
rYB-1
rDbpA_a+b
130
100
75
55
35
endo DbpA
rec DbpA_a
rec YB-1
rec DbpA_b
endo. YB-1
Flag
YB-1 C-Term
DbpA C-Term

Figure S4. Purification of recombinant human DbpA_a and DbpA_b proteins. (A) Plasmids containing Flag-tagged recombinant human DbpA_a or DbpA_b were transfected into HEK293 cells. After 48 hours, the cells are lysed and the recombinant protein purified by affinity chromatography, as previously described [28]. To visualize the purity, aliquots of each fraction were separated on NuPAGE gels and stained with Coomassie brilliant blue dye. (B) For western blotting, the gels were transferred onto PVDF membranes and the Flag-epitope tag detected. (C) Aliquots of purified recombinant proteins were blotted using the indicated antibodies. The position of recombinant DbpA_a, DbpA-b (r DbpA_a, rDbpA-b) is indicated 65 and 50 kDa by the broken black lines. rYB-1 is indicated by the broken blue line at 55 kDa. The endogenous YB-1 band (eYB-1) is indicated by the broken red line at 50 kDa. Endogenous DbpA appears at 130 kDa.
